# The Positive Effect of Workplace Accommodation on Creative Performance of Employees With and Without Disabilities

**DOI:** 10.3389/fpsyg.2020.01217

**Published:** 2020-06-17

**Authors:** Xiangyu Man, Xiji Zhu, Cong Sun

**Affiliations:** ^1^Central University of Finance and Economics, Beijing, China; ^2^The Chinese University of Hong Kong, Shenzhen, Shenzhen, China

**Keywords:** workplace accommodation, employees with disabilities, employees without disabilities, creative performance, disability

## Abstract

The issue of workplace accommodation is vital to employees with and without disabilities, as well as employers and organizations. Drawing on the self-efficacy theory, this paper examines the mechanism and contingency of the relationship between workplace accommodation and employee creative performance. Specifically, we argue that creative self-efficacy is the key factor through which workplace accommodation promotes employee creative performance. Aligning with the identity-blind diversity management, we hold a continuous view of disability that everyone has a certain level of disability ranging from zero to a high level of disability severity. Disability severity moderates the relationship between workplace accommodation and creative self-efficacy, and the aforementioned indirect effect, such that the positive relationship and the indirect effect are stronger for employees with a lower level of disability severity. Data collected from a multi-wave multisource field study with 300 participants provide general support for our hypotheses. This research contributes to the literature by (a) providing empirical support for the identity-blind diversity management, (b) extending the research on the psychological well-being and performance of employees with disabilities, and (c) enlarging the nomological network of workplace creativity. Practically, our research provides insights for practitioners to promote workplace accommodation practices, as workplace accommodation is not only essential for including employees with disabilities but also helpful in boosting the creative performance of all employees.

## Introduction

With an aging workforce and the equal opportunities workplace movement, an increasing number of people with disabilities (PWD) enter workplaces (Zhu et al., 2019). As an important diversity attribute in the workplace, the employer diversity management strategies regarding disability have been stagnant at the identity-conscious approach for a long time (Gould et al., 2020). The identity-conscious approach of diversity management toward PWD labels certain employees with disability identities and designs corresponding management programs, such as workplace accommodation, to take care of their needs. A workplace accommodation is defined as “modifications in the job, work environment, work process, or conditions of work that reduce physical and social barriers so that people with disabilities experience equal opportunity in a competitive work environment” (Colella and Bruyère, 2011, p. 478). Taking the identity-conscious perspective, employers assume that employees with disabilities are of lower status in the organization and are victims of discrimination and stigmatization. They reactively adopt reasonable accommodations for PWD to fulfill the legal requirement and minimize the inferior status of employees with disabilities.

While workplace accommodation is deemed as a key organizational practice to realize the full employment and equal opportunity for PWD (Baldridge and Swift, 2013), its sole focus on PWD with the identity-conscious approach impedes the knowledge of its effects on other important stakeholders such as their coworkers without disabilities. Scholars begin to call for a transition from the sole focus on PWD with the identity-conscious approach to a broader focus on all employees with the identity-blind approach (Schur et al., 2014). The identity-blind approach posits that organizations should focus more on the integration of all diverse groups to provide them with equal and inclusive environments. Following this approach, Schur et al. (2014) argue that workplace accommodation can be viewed “in the broader context of accommodating all employee needs.” In the workplace, not only employees with disabilities ask for workplace accommodation to better perform in the job but also the older workers, pregnant women, and employees with religious needs and with family responsibilities need workplace accommodations such as flexible working schedules and family-friendly programs. However, most of the current studies have been focused on the positive effects of workplace accommodation for PWD solely or the cost–benefit analysis for employers (see Nevala et al., 2015, for a systematic review) and less explored the positive effects of workplace accommodation for employees without disabilities. This lack of knowledge prohibits the promotion of workplace accommodation in organizations and limits the utilization and development of the human capital of employees with and without disabilities.

In light of this problem, this paper adopts the identity-blind approach and investigates the effect of workplace accommodation on all employees regardless of their disability identities. Following the pioneering research of Schur et al. (2014) on the broader definition of workplace accommodation and the current trend of focusing on identity-blind employee inclusion, we define workplace accommodation as modifications in the job, work environment, work process, or conditions of work that reduce physical and social barriers for all the employees. We choose creative performance as our key dependent variable because creativity has been confirmed as a major benefit brought by diversity (e.g., Kurtzberg, 2005; Miron-Spektor et al., 2011), and disability is regarded as an important domain of diversity (Dovidio et al., 2011; Moore et al., 2011; Dwertmann, 2016). Drawing on the self-efficacy theory (Bandura, 1977), we elaborate on how workplace accommodation influences employee creative performance through creative self-efficacy.

**FIGURE 1:**
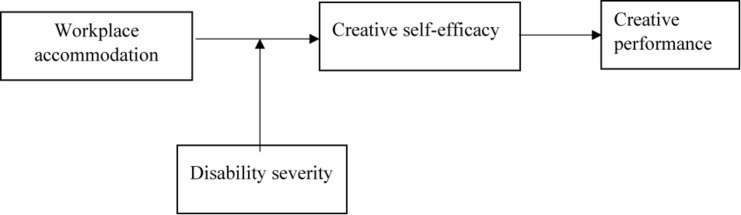
Research model.

Following the Law of the People’s Republic of China on Protection of Disabled Persons (2008) which is consistent with the definition of the United Nations Convention on the Rights of Persons with Disabilities, we define disability as “loss or abnormality of a certain organ or function, psychologically or physiologically, or in anatomical structure and lost wholly or in part the ability to perform an activity in a normal way” (Zhu et al., 2019, p. 22). People with disabilities refer to those who fulfill the above definition and hold the government-issued disability certificate in China. The certificate issued by the government also indicates the disability severity of a certain type of disability, ranging from a low-level disability severity (25–50% functional loss) to a high-level disability severity (above 50% functional loss). Based on a continuous view of disability that everyone has a certain level of disability ranging from zero to a high level of disability severity, we further propose that disability severity will moderate the relationship between workplace accommodation and creative self-efficacy, as well as the indirect effect of workplace accommodation on creative performance through creative self-efficacy. Figure 1 presents our conceptual model.

Our study attempts to make two major contributions to the extant literature. First, we contribute to the current workplace accommodation literature by redefining it from the identity-blind approach. With the identity-conscious approach, the use of workplace accommodation for PWD leads researchers to narrowly focus on its positive effect for PWD (e.g., Cleveland et al., 1997; Nevala et al., 2015), overlooking its potential benefits to others. By developing and validating a new workplace accommodation scale for both employees with and without disabilities, this study tries to demonstrate the positive effect of workplace accommodation on the creative performance of all employees. By doing so, this study expands the positive effect of workplace accommodation for a broader scope of employees rather than merely PWD.

Second, our study contributes to diversity management literature by framing workplace accommodation as an identity-blind strategy and investigating its effect. Compared with traditional identity-conscious diversity management strategies (e.g., affirmative actions; see Leslie et al., 2014, for a review), it is still unclear how identity-blind diversity management strategies operate. Without a good understanding of the mechanism, practitioners would doubt the effectiveness of identity-blind diversity management. In line with this practical demand, our study tries to reveal how workplace accommodation, as a kind of identity-blind diversity management initiative, increases the creative performance of all employees from the self-efficacy perspective.

## Theory and Hypotheses

### Workplace Accommodation and Creative Performance

In this paper, we define workplace accommodation as modifications in the job, work environment, work process, or conditions of work that reduce physical and social barriers for all employees. When employees perceive a high level of workplace accommodation in the organization, they are able to participate in most formal and informal organizational activities, and they contribute their talents such as creativity, thereby realize their self-worth in the organization.

We argue that workplace accommodation research should follow the switch from an identity-conscious to identity-blind orientation in the diversity literature (Konrad and Linnehan, 1995; Roberson, 2006). An identity-conscious approach to diversity refers to the orientation that minorities with different identities should be cared for, and special organizational practices should be designed to tailor for the special needs of these employees. Under this approach, affirmative action programs would be initiated to protect the benefits and well-being of minority groups in the organization.

However, as Thomas (1990) put it, affirmative action is dying out as a natural death. He believed that organizations should affirm diversity rather than affirm their actions toward minorities. This is referred to as the identity-blind approach to employee diversity. The identity-blind approach emphasizes that the same human resource management policy should be imposed on all employees, regardless of their identities and characteristics (Richard and Johnson, 2001). Diversity should be considered as an asset to the organization. Managers only need to care that all employees are equally included and assimilated in their organization while maintaining their identities. Thus, following this idea, we argue that workplace accommodation should be adopted toward all the employees and it can help organizations harvest the benefits of diversity.

We argue that workplace accommodation can increase employee creative performance. Creativity is defined as the production of novel and useful ideas by an individual or small group of individuals working together (Amabile, 1988, p. 124). Following Amabile (1988) and Zhou and George (2001), we define creative performance as the generation of novel and useful ideas concerning organizational products, services, and other issues that are beneficial to the organizations. Amabile (1983, 1988) componential model of individual creativity, which is the most influential framework to examine the factors affecting creativity, outlined three major components necessary for individual creativity in any given domain: motivational components such as intrinsic task motivation, domain-relevant skills (expertise, resources), and creativity-relevant skills (techniques). As stressed by Amabile (1996), the most important premise of this theory is that work environments have an impact on creativity by affecting the motivational and skill-related components (task-relevant and creativity-relevant skills) that contribute to individual creativity. Amounts of empirical evidence have been shown to support the componential model of individual creativity, and the role of a motivational component of the individual creativity theory has received a greater weight of investigation than the other two (Shalley et al., 2004; Zhou and Shalley, 2010; Anderson et al., 2014). There is also some empirical evidence showing that a favorable work environment can enhance creative performance (e.g., Zhou and George, 2001). Following this logic, we argue that workplace accommodation, which creates a more favorable work environment, can increase employee creative performance at work.

Hypothesis 1. Workplace accommodation is positively associated with creative performance.

### The Mediating Role of Creative Self-Efficacy

While workplace accommodation should be generally preferable to an organization, little is known about the mechanism through which workplace accommodation would affect employee outcomes. It is the purpose of this study to investigate how workplace accommodation would affect the creative performance of employees. We argue that creative self-efficacy might be the major mechanism.

Based on the self-efficacy theory (Bandura, 1997) and the literature of creative self-efficacy (Tierney and Farmer, 2002), creative self-efficacy, as a specific, state-like self-efficacy, is defined as an employee’s confidence in the ability to complete the creative tasks or complete the job creatively (Tierney and Farmer, 2002). It is a key motivation in achieving creative performance (Ford, 1996; Bandura, 1997; Tierney and Farmer, 2002). Moreover, creative self-efficacy is widely proved to be positively related to creative performance in the previous literature (e.g., Gong et al., 2009; Tierney and Farmer, 2011).

Drawing from self-efficacy theory (Bandura, 1977), we argue that workplace accommodation is positively associated with creative self-efficacy, thus it can increase creative performance through increased creative self-efficacy. Self-efficacy theory stated that self-efficacy is constructed from four principal sources of information: enactive mastery experience, vicarious modeling, verbal persuasion, and arousal. We argue that workplace accommodation is related to all four mechanisms of creative self-efficacy development, thus leading to a high creative self-efficacy. By modifying the job, work environment, work process, or conditions of work, workplace accommodation can reduce physical and social barriers and create more learning opportunities in the work environment, increasing enactive mastery, vicarious modeling, verbal persuasion, and arousal.

First, workplace accommodation can promote enactive mastery experience. Employees who are well accommodated will get more resources and support to help them complete creative job tasks successfully, which in turn will bring more enactive mastery experience to strengthen creative self-efficacy. Second, workplace accommodation offers the chance for vicarious modeling. Employees who are well accommodated will have better opportunities to observe successful role models, especially those who are similar to them. This is especially true for those workgroup members who are exposed to multiple skillful coworkers (Bandura, 1997). The opportunity to learn from different coworkers as role models offered by the inclusive environment will enhance the confidence in team members’ efficacy. Third, getting exposure to the social interactions in the organization will facilitate more social persuasion as a source to increase creative self-efficacy. When employees are well accommodated in the organization, they will feel free to speak and listen, and more feedback and encouragement will be given to them to help boost their creative self-efficacy. Finally, workplace accommodation can activate positive physiological and affective states to enhance creative self-efficacy. When employees feel well accommodated, they feel included and connected with the groups without losing their uniqueness. They will feel safer and less anxious, and hence their creative self-efficacy will be enhanced. Based on the above, we hypothesize the following:

Hypothesis 2. Workplace accommodation has a positive indirect effect on creative performance through creative self-efficacy, such that workplace accommodation is positively related to creative self-efficacy, and creative self-efficacy is positively associated with creative performance.

### The Moderating Role of Disability Severity

While the four sources (enactive mastery, vicarious modeling, verbal persuasion, and arousal) to enhance efficacy provided by workplace accommodation will lead to the increase of creative self-efficacy in general, they may not be used equally well to construe creative self-efficacy by employees. Employees process the sources of efficacy selectively, depending on their attributes and the specific situations in which they are embedded (Gist and Mitchell, 1992). Following the self-efficacy theory’s person–environment interaction argument that self-efficacy is predicted from both aspects of the social environment and individual differences (Bandura, 1977, 1997), we posit that the positive effect of workplace accommodation on creative self-efficacy is moderated by disability severity. We argue that employees with a less severe disability will benefit more from the positive effect of workplace accommodation on creative self-efficacy through the four sources of self-efficacy.

First, employees with a lower level of disability severity will have more enactive mastery experiences triggered by workplace accommodation. Previous research found that employees without disabilities have more knowledge exposure and more job self-efficacy in general than employees with disabilities (Stone and Colella, 1996; Colella and Bruyère, 2011; Zhu et al., 2019). In this paper, we hold a continuous view of disability and view employees without disabilities as a low level of disability severity. For employees with a lower level of disability severity, who start with more knowledge exposure and self-efficacy than those with a higher level, workplace accommodation will let them know more information about the task and clearer beliefs of what level of difficulties they can achieve. We argue that workplace accommodation for employees with a lower level of disability severity would result in more mastery experiences to build their efficacy than others with a higher level of disability severity.

Second, employees with a lower level of disability severity will have more vicarious modeling experiences provided by workplace accommodation. Workplace accommodation can provide all the employees with an inclusive environment to acquire, share, and integrate the knowledge of coworkers, reinforcing their beliefs in the magnitude of their efficacy through vicarious learning. Self-efficacy theory stated that the learning outcome is affected by the attributes of the observers, the model, and the learning situations such as the similarity between observer and model (Bandura, 1977, 1997). Employees with disabilities are usually stigmatized and have a low status in the workplace, and there are less successful models for employees with disabilities than employees without disabilities (Zhu et al., 2019). Thus, the lack of numbers of successful models and the dissimilarity between the learner and model diminishes the learning effectiveness of employees with a high disability severity.

Moreover, employees with a lower level of disability severity will also get more social persuasion and generate more positive physiological and affective states provided by workplace accommodation. There is a long-existing low competence stereotype toward PWD (Fiske et al., 1999). Employees with a lower level of disability will suffer less from this negative stereotype and thus get more social persuasion from others when they get well accommodated in the workplace. Meanwhile, employees with disabilities also tend to have self-stigma of low competence due to discrimination from others (Zhu et al., 2019). Employees with a higher level of disabilities will likely be less thriving at work and have less positive physiological and affective states to foster self-efficacy beliefs. Based on these arguments, we hypothesize that:

Hypothesis 3. Disability severity moderates the relationship between workplace accommodation and creative self-efficacy, such that the positive relationship is stronger for employees with a lower level of disability severity.

With previous hypotheses in place, we propose a moderated mediation model whereby workplace accommodation influences employee creative performance *via* creative self-efficacy, with disability severity moderating the first-stage relationship. We argue that workplace accommodation enhances creative self-efficacy, and creative self-efficacy, in turn, increases creative performance. As employees with lower levels of disability severity have more resources to get the enactive mastery, vicarious modeling, verbal persuasion, and arousal sources to build creative self-efficacy, we expect that this positive indirect effect would be stronger for employees with a lower level of disability severity. Thus, we propose:

Hypothesis 4. Disability severity moderates the indirect effect of workplace accommodation on creative performance through creative self-efficacy, such that the positive indirect effect is stronger for employees with a lower level of disability severity.

## Study 1: Scale Development of Workplace Accommodation

### Item Generation

We generated the items of workplace accommodation based on an intensive literature review and an in-depth interview with the human resource (HR) manager in the pilot company. First, there is no established workplace accommodation scale for all the employees. Based on Colella and Bruyère’s (2011) classification of different categories of workplace accommodation, we interviewed the HR manager in the pilot study and discussed the most common practices in the company to guarantee our content validity. Integrating the content of Colella and Bruyère (2011) workplace accommodation classification and the feedback of the HR manager, we finally generated five items for workplace accommodation. The five items were “the entrance of the company has a ramp or automatic doors to facilitate all employees,” “the company has sufficient internal accessibility to facilitate all employees,” “I can use the adaptive tools such as ergonomic table and chair in my work,” “When necessary, I can flexibly adjust working hours according to my physical condition,” “I think the current work environment for me is barrier-free and convenient.”

### Pilot Sample

To validate the scale, we conducted a pilot study in a sample similar to those who would be included in the hypothesis testing study. The sample was from a manufacturing company located in northern China. We surveyed 293 participants on their perceived workplace accommodation (on a seven-point Likert-type scale) and demographic background. Among the participants, 164 were with disabilities and the other 129 were without disabilities; 51% were male; the average age was 32.11 years (SD = 7.14); the average tenure was 62.54 months (SD = 28.03).

### Exploratory Factor Analysis

To utilize the pilot data efficiently, we randomly split the 293 participants into two subsamples, one with 140 participants for conducting exploratory factor analysis (EFA), and the other 153 participants for conducting confirmatory factor analysis (CFA).

An EFA (*N* = 140) of workplace accommodation items using varimax rotation produced one factor, and all item loadings were greater than 0.73 (as shown in Table 1), indicating a good factor structure (Tabachnick and Fidell, 2007). The factor explained 64.04% of the variance. The Cronbach’s alpha coefficient for the scale was 0.85, and all the inter-item correlations were above 0.38, indicating satisfactory reliability. Moreover, as shown in Table 1, the factor loadings of the items did not differ significantly across employees with and without disabilities. As indicated by Schur et al. (2014), the types of accommodation needs and requests of employees with and without disabilities were similar in the workplace. Thus, the scale has a satisfactory level of ecological validity.

**TABLE 1:** EFA factor loadings of perceived accommodation items^a^.

Item	Full sample	Sample with disabilities	Sample without disabilities
The entrance of the company has a ramp or automatic doors to facilitate all employees.	0.79	0.83	0.74
The company has sufficient internal accessibility to facilitate all employees.	0.81	0.77	0.87
I can use the adaptive tools such as ergonomic table and chair in my work.	0.83	0.81	0.86
When necessary, I can flexibly adjust working hours according to my physical condition.	0.73	0.75	0.74
I think the current work environment for me is barrier-free and convenient.	0.83	0.80	0.87

*^a^n = 140. EFA, exploratory factor analysis.*

### Confirmatory Factor Analysis

We conducted CFA using another subsample (*N* = 153) to provide further evidence of the scale of workplace accommodation. In this sample, the Cronbach’s alpha coefficient was 0.85, indicating good reliability. In the CFA, we loaded five items onto one latent factor, the model indicated a satisfactory fit: χ^2^[5] = 42.05, comparative fit index (CFI) = 0.90, standardized root mean square residual (SRMR) = 0.065, the root mean square error of approximation (RMSEA) = 0.22. Given that our model is a small degree of freedom (df) model with a small sample size, CFI is more appropriate to estimate the model fit than RMSEA (Kenny et al., 2015).

## Study 2: Hypothesis Testing

### Participants and Procedures

We collected data from a medical equipment company in northern China. This company was chosen because it was recognized as a disability-friendly company and provides employees with workplace accommodation. With the help from the human resource department of the company, survey questionnaires were distributed in person to all the 464 employees. There are 78 employees holding licenses of disability, accounting for 16.8% of all the 464 employees in the company. Our research was approved by the research ethics committee in our universities, and the research participants provided their written informed consent to take part in the study.

We collected data from both employees and supervisors in three waves to control for the common method variance (Podsakoff et al., 2003). In the first wave, we collected data on workplace accommodation and demographic data including disability severity, gender, age, tenure, and education from the employees. Two weeks later, we collected data on creative self-efficacy from the employees. In the final wave (2 weeks after the second wave data collection), we collected creative performance rated by supervisors. After each wave of the survey, each participant received a small gift as a token of appreciation.

Out of 464 respondents, 300 employees provided complete data for analyses after listwise deletion. We only included the completed and matched data in our final sample. Out of the 300 participants, 78% were female, 65% were educated in middle school or below, and 19% were employees with disabilities. The average age was 28.94 years (SD = 6.59), and the average organization tenure was 32.21 months (SD = 33.43).

### Measures

#### Workplace Accommodation

We measured workplace accommodation by a five-item scale created for this study. The scale was used on a 7-point Likert-type scale, and the Cronbach’s alpha coefficient was 0.75.

#### Disability Severity

We coded disability severity in a continuous way (0–4) according to whether the investigated employees have a certificate issued by local government and indicates the disability severity of a certain type of disability. Here, 4 represents the highest level of disability, whereas 0 represents no disability.

#### Creative Self-Efficacy

We measured creative self-efficacy using a three-item scale developed by Tierney and Farmer (2002) on a 7-point Likert-type scale. An example item was, “I have confidence in my ability to solve problems creatively.” Cronbach’s alpha coefficient for this scale was 0.87.

#### Creative Performance

We used the 13-item creativity scale developed by Zhou and George (2001) to measure individual creative performance. Employees’ supervisors rated their creative performance using a 7-point Likert-type scale. An example item was, “this employee suggests new ways to increase quality.” Cronbach’s alpha coefficient for this scale was 0.97.

#### Control Variables

We also measured gender (0 = male, 1 = female), age (in years), tenure (in months), education (1 = junior middle school and below, 2 = senior middle school, 3 = college, university, and above) of the employees as control variables.

### Descriptive Statistics

Table 2 presents the means, standard deviations, and zero-order Pearson correlations of studied variables. As shown in the table, workplace accommodation was positively correlated with creative self-efficacy (*r* = 0.12, *p* < 0.05) and not significantly correlated with creative performance [*r* = –0.03, not significant (*n.s.*)]. In addition, creative self-efficacy was positively correlated with creative performance (*r* = 0.19, *p* < 0.01).

**TABLE 2:** Means, standard deviations, and correlations^a^.

	Variables	Mean	SD	Skewness	Kurtosis	1	2	3	4	5	6	7
1.	Age (years)	28.94	6.59	0.24	–0.38							
2.	Tenure (months)	32.21	33.43	1.56	2.65	0.37**						
3.	Education^b^	1.50	0.73	0.74	–0.81	−0.18**	–0.11					
4.	Gender^c^	0.78	0.41	–1.37	–0.14	0.14*	−0.20**	−0.13*				
5.	Workplace accommodation	5.43	1.02	–0.55	0.05	–0.06	–0.08	0.12*	–0.02			
6.	Disability severity	0.39	0.91	2.55	5.58	–0.02	0.25**	−0.15*	−0.31**	0.04		
7.	Creative self-efficacy	5.03	1.05	–0.22	0.35	0.26**	0.11	0.21**	–0.10	0.12*	-0.07	
8.	Creative Performance	4.46	1.15	–0.29	–0.10	0.13*	0.16**	0.12*	−0.12*	–0.03	0.08	0.19**

^a^n = 300; values in parentheses on the diagonal are Cronbach’s alpha coefficients. ^b^Education coded as 1 = Junior Middle School and Below, 2 = Senior Middle School, 3 = College, University, and Above. ^c^Gender coded as 0 = Male, 1 = Female. *p < 0.05, **p < 0.01, two-tailed tests.

### Testing the Main and Indirect Effects

We conducted hierarchical multiple regression analysis to test the Hypotheses, entering the control variables, the independent variable (workplace accommodation), moderator (disability severity), mediator (creative self-efficacy), and the interaction term on separate steps. Hypothesis 1 predicts that workplace accommodation is positively associated with creative performance; as shown in Table 3 (Model 2), the regression coefficient of creative performance on workplace accommodation was not significant (β = –0.03, *n.s.*). Thus, Hypothesis 1 was not supported. Hypothesis 2 predicts that workplace accommodation has a positive indirect effect on creative performance through creative self-efficacy; as shown in Table 3 (Model 4), workplace accommodation was not significantly related to creative performance (β = –0.06, *n.s.*), with creative self-efficacy included in the regression model, which was significantly associated with creative performance (β = 0.17, *p* < 0.05). We used bootstrap analyses to test the indirect effect (Edwards and Lambert, 2007), generating 1,000 samples and computing bias-corrected confidence intervals. The results indicated a significant indirect effect of workplace accommodation on creative performance *via* creative self-efficacy [*indirect effect* = 0.02; the 95% confidence interval of the indirect effect was (0.001, 0.054)]. Thus, Hypothesis 2 was supported.

**TABLE 3:** Hierarchical multiple regression results predicting creative performance^a^.

Variables	Model 1	Model 2	Model 3	Model 4	Model 5	Model 6	Model 7
**Control**							
Age (years)	0.02	0.02	0.01	0.01	0.02*	0.02*	0.02
Tenure (months)	0.00	0.00	0.00	0.00	0.00	0.00	0.00
Education^b^	0.21*	0.22*	0.16	0.17	0.23*	0.24*	0.19*
Gender^c^	–0.26	–0.26	–0.22	–0.22	–0.20	–0.21	–0.15
**Independent**							
Perceived workplace accommodation		–0.03		–0.05	–0.03	–0.04	–0.06
**Moderator**							
Disability severity					0.16	0.14	0.16
**Interaction**							
Perceived workplace accommodation × Disability severity						0.10	0.13
**Mediator**							
Creative self-efficacy			0.14*	0.15*			0.17*
*R*^2^	0.06	0.06	0.07	0.08	0.06	0.07	0.09
Δ*R*^2^	0.06**	0.00	0.02*	0.02*	0.00	0.01	0.02*
*F*	4.69**	3.79**	4.73**	4.03**	3.28**	3.15**	3.60**
Δ*F*	4.69**	0.25	4.62*	4.94*	0.72	2.29	6.36*

^a^n = 300; unstandardized coefficients are reported. ^b^Education coded as 1 = Junior Middle School and Below, 2 = Senior Middle School, 3 = College, University, and Above. ^c^Gender coded as 0 = Male, 1 = Female. *p < 0.05, **p < 0.01, two-tailed tests.

### Testing the Moderation and Moderated Mediation

**FIGURE 2:**
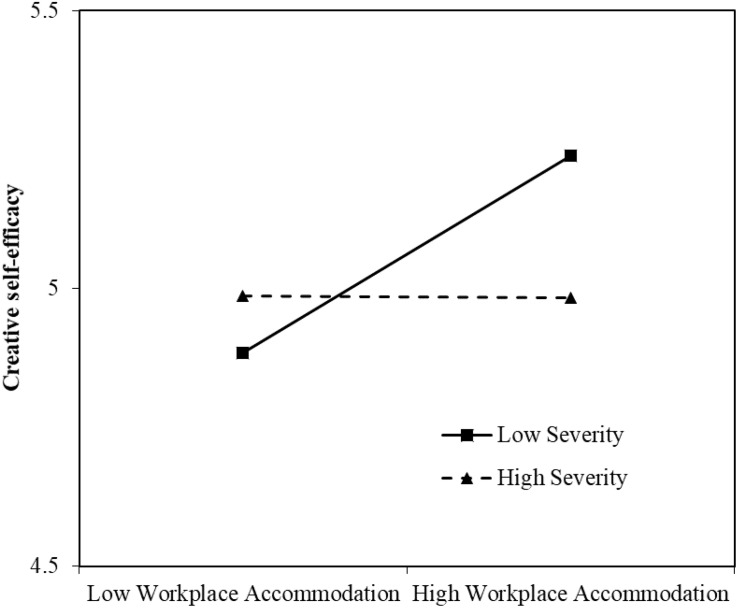
Plot of interaction predicting creative self-efficacy from workplace accommodation and disability severity.

Hypothesis 3 predicts that disability severity moderates the relationship between workplace accommodation and creative self-efficacy such that the relationship is positive and stronger for employees with a lower level of disability severity. As shown in Table 4 (Model 4), the interaction between workplace accommodation and disability severity was negatively related to creative self-efficacy (β = –0.14, *p* < 0.05). We then plotted the interaction effects using Aiken and West (1991)’s procedure, computing slopes for employees with a low disability severity (+1 SD; severity = 0) and a high disability severity (–1 SD; severity = 1.30). Figure 2 shows the interaction pattern. Specifically, workplace accommodation was positively related to creative self-efficacy for employees with a low level of disability severity (β = 0.17, *p* < 0.01) but was unrelated to creative self-efficacy for employees with a high level of disability severity (β = –0.00, *n.s.*). Thus, Hypothesis 3 was supported.

**TABLE 4:** Hierarchical multiple regression results predicting creative self-efficacy^a^.

Variables	Model 1	Model 2	Model 3	Model 4
**Control**				
Age (years)	0.05**	0.05**	0.05**	0.05**
Tenure (years)	–0.00	0.00	0.00	0.00
Education^b^	0.36**	0.34**	0.32**	0.32**
Gender^c^	–0.28	–0.27	−0.33*	−0.31*
**Independent**				
Perceived workplace accommodation		0.11*	0.12*	0.12*
**Moderator**				
Disability severity			–0.10	–0.06
**Interaction**				
Perceived workplace accommodation × Disability severity				−0.14*
*R*^2^	0.15	0.16	0.16	0.18
Δ*R*^2^	0.15**	0.01*	0.00	0.02*
*F*	12.70**	11.07**	9.59**	9.06**
Δ*F*	12.70**	4.06*	1.99	5.09*

^a^n = 300; unstandardized coefficients are reported. ^b^Education coded as 1 = Junior Middle School and Below, 2 = Senior Middle School, 3 = College, University, and Above. ^c^Gender coded as 0 = Male, 1 = Female. *p < 0.05, **p < 0.01, two-tailed tests.

The moderated mediation prediction in Hypothesis 4 requires tests of whether the indirect effect of workplace accommodation on creative performance varies as a function of disability severity. We tested this moderated mediation hypothesis using moderated path analysis (Edwards and Lambert, 2007). As shown in Table 5, the size of the difference in the indirect effect of workplace accommodation on creative performance was 0.03 (*p* < 0.05), with the 95% confidence intervals computed using the bootstrap estimates excluding zero. Specifically, the indirect effect of workplace accommodation on creative performance was positive for employees with a low disability severity (*indirect effect* = 0.03, *p* < 0.05) while not significant for employees with a high disability severity (*indirect effect* = –0.00, *n.s.*). Therefore, the indirect effect was significantly stronger for employees with a low disability severity. Thus, Hypothesis 4 was supported.

**TABLE 5:** Moderated path analysis results^a^.

	Workplace accommodation (X) → creative self-efficacy (M) → creative performance (Y)				
Paths^b^	First stage P_MX_	Second stage P_YM_	Direct effects P_YX_	Indirect effects P_YM_ P_MX_	Total effects P_YX_ + P_YM_ P_MX_
Severity = 0	0.12*	0.17*	–0.11	0.03*	–0.08
Severity = 1.30	–0.06	0.17*	0.05	–0.00	0.05
Differences	0.18*	0.00	–0.16	0.03*	–0.13

^a^n = 300; tests of differences for the indirect and total effects were based on bias-corrected confidence intervals derived from bootstrap estimates. ^b^P_MX_ is the path from workplace accommodation to creative self-efficacy; P_YM_ is the path from creative self-efficacy to creative performance; P_YX_ is the path from workplace accommodation to creative performance. *p < 0.05, two-tailed tests.

## Discussion

The present study adopted the identity-blind approach of diversity management and investigated the effect of workplace accommodation on the creative performance of employees with a full range of disability severity. Using a sample of 300 employees in China, we found that workplace accommodation promotes employee creative performance by increasing their creative self-efficacy. Our research confirmed that employee creative self-efficacy is a key intervening mechanism linking employee perceived workplace accommodation and their creative performance. The results of our study also implied that disability severity moderated the relationship between workplace accommodation and creative self-efficacy, such that the relationship is stronger for employees with a lower level of disability severity.

### Theoretical Implications

Our findings provide new insights into the relationships among workplace accommodation, creative performance, creative self-efficacy, and disability severity, thus having several theoretical implications. First, in particular, our study shows that using an identity-blind diversity management strategy can yield positive workplace outcomes. Specifically, workplace accommodation helps boost employee creative performance by enhancing creative self-efficacy. This finding reveals how and why workplace accommodation benefits the organization, providing empirical support for the identity-blind approach in understanding and promoting workplace diversity and inclusion.

Second, in response to the call for learning more about employees with disabilities experience to help them increase their psychological well-being and performance in organizations (Colella and Varma, 2001; Colella and Bruyère, 2011), we examine whether the effects of workplace accommodation is contingent on employees’ disability levels. We find that disability severity moderates the positive relationship between workplace accommodation and creative self-efficacy, such that the relationship is stronger for employees with a lower level of disability severity. By focusing on the psychological experience of employees with different levels of disability severity, we bring a new internal and identity-blind perspective to future studies on the influence of organizational practices toward the treatment of all the employees in the workplace with disability diversity.

Third, this study also contributes to creativity literature by introducing workplace accommodation as a possible facilitator. Our findings demonstrate the positive indirect effect of workplace accommodation through creative self-efficacy, expanding our knowledge of environmental factors that would facilitate the employee creative performance. Moreover, Amabile (1983, 1988, 1996) componential framework of creativity sets the stage for investigating individual creativity in the motivational approach which attracts most research attention in organizational creativity compared to the other approaches such as cognitive approach and affective approach (Zhou and Shalley, 2010). Our study demonstrates that the motivational factor creative self-efficacy is a key mechanism linking the environment variable (workplace accommodation) and creativity outcome (creative performance). Furthermore, our study finds that there is no significant direct effect of workplace accommodation on creative performance, indicating there may be other oppression mechanisms underlying this relationship.

### Practical Implications

Our study provides several practical implications for the emerging practices of promoting workplace accommodation strategies in managing diversity. First, our findings offer practitioners evidence for the real effect of the identity-blind management of diversity. With a sound understanding of the operating mechanisms of workplace accommodation, the current movement of identity-blind diversity management practices in organizations may be propagated, especially in developing areas such as China.

The issue of workplace accommodation has generated a great deal of attention in the past few years after the passage of the Americans with Disabilities Act (ADA) in 1990. Most current workplace accommodation research and practices are western-based. In 2006, the United Nations adopted the Convention on the Rights of Persons with Disabilities (CRPD) to promote reasonable accommodation including workplace accommodation for PWD across the world (CRPD, Article 2). China is among the first countries which signed CRPD. However, the Chinese government has not defined workplace accommodation officially and has not added any accommodation requirements in the law. Thus, the current practices and research in China are far behind and mainly follow the western and UN’s definitions and practices of workplace accommodation. By investigating the effect of workplace accommodation for employees in China, this paper hopefully can facilitate proactive workplace accommodation in China.

Second, our findings imply that there may be some pitfalls in identity-blind diversity management practices. Echoed with Leslie’s model of unintended consequences of diversity initiatives (Leslie, 2019), we demonstrate that workplace accommodation benefit employees with lower levels of disability severity more than those with higher levels of disability severity. This is due to the creative self-efficacy difference between the groups. Thus, organizations should pay attention to close this creative self-efficacy gap through decreasing the discrimination toward employees with high levels of disability (Colella et al., 2017) and cultivating a more favorable climate for inclusion (Nishii, 2013). Moreover, the creative self-efficacy gap may be enlarged by the difference in the training for self-efficacy as self-efficacy can be fostered through appropriate training programs (Gist and Mitchell, 1992; McNatt and Judge, 2008; Reeves et al., 2011). Thus, employees, especially those with high levels of disability severity, should be provided with training which directly enhances through the utilization of mastery, modeling, and persuasion experiences of their capabilities or understanding of how to use skills successfully in dealing with workplace accommodation issues (McAuley et al., 1999; Grey, 2013; Ouweneel et al., 2013).

### Limitations and Suggestions for Future Research

Despite the consistency found in our tested model, our study has several limitations. In drawing attention to these limitations, we are also suggesting directions for future research. First, although we collect our independent variable 1 month before the dependent variable, causal relationships cannot be inferred because of the cross-sectional nature of our studies. Rigorous causal relationship research design such as experiment and longitudinal studies are encouraged to verify the causal relationship in our model. Second, although we adopted some methods to reduce the odds for common method bias to influence the study results, such as temporally separated the measurement of the independent variable and the moderator from that of the dependent variable, collected data from different sources (employees and supervisors), there are still some statistical concerns which may harm our statistical validity. For example, our sample is relatively small, and our finding is only based on the sample collected in one single country. As the widely existing cross-cultural difference, more evidence from different countries should be shown to support the generalizability of our results. Finally, future studies should analyze curvilinear relationships and consider other potential moderating variables to further develop our model. For example, mindfulness may play a role in moderating the effects of the organizational environment such as workplace accommodation (e.g., Montani et al., 2019).

## Conclusion

The issue of workplace accommodation is vital to employees with and without disabilities, as well as employers and organizations. Drawing on the self-efficacy theory, this study explored the connections between workplace accommodation and employee creative performance. By applying a novel and broader view of workplace accommodation to predict its effects on employees with a full range of disability severity, we believe that our study demonstrates the availability and importance of workplace accommodation as an identity-blind diversity management strategy. We hope our work will lead to a broader exploration of workplace accommodation in the diversity management research and contribute to the promotion of workplace accommodation practices, maximizing the utilization of all employees’ talents and abilities.

## Data Availability Statement

The datasets generated for this study are available on request to the corresponding author.

## Ethics Statement

The studies involving human participants were reviewed and approved by The research ethics committee in CUFE Business School. The patients/participants provided their written informed consent to participate in this study.

## Author Contributions

All authors contributed to the manuscript and approved the submitted version.

## Conflict of Interest

The authors declare that the research was conducted in the absence of any commercial or financial relationships that could be construed as a potential conflict of interest.
